# Can sports consumption cities reduce residents' medical expenditures? Evidence from China

**DOI:** 10.3389/fpubh.2026.1822237

**Published:** 2026-04-13

**Authors:** Ziwen Zhang, Meichao Cheng, Jingting Ni, Shun Wang

**Affiliations:** 1 School of Physical Education, Huai Bei Normal University, Huaibei, Anhui, China; 2 School of Physical Education, Shandong University, Jinan, Shandong, China; 3 Department of Sport and Leisure Studies, Namseoul University, Cheonan-si, Republic of Korea

**Keywords:** CFPS, China, difference-in-difference, medical expenditures, pilot, sports consumption cities

## Abstract

**Background:**

The relentless rise in healthcare expenditures poses a severe challenge to the sustainable development of global societies. In China, a pilot program for Sports Consumption Cities (SCC) represents a pioneering strategic effort to align growth in the sports industry with public health promotion. This study aims to examine the causal impact of this city-level non-medical intervention on residents' medical expenditures and to elucidate how active lifestyle policies function as a cost-control strategy.

**Methods:**

Using longitudinal data from the China Family Panel Studies (CFPS) from 2014 to 2022, we employed a Difference-in-Differences (DID) strategy to evaluate the impact of SCC construction on residents' medical expenditures. Additionally, a mediation model was applied to examine four parallel pathways: sports consumption, physical exercise frequency, self-rated health, and depression levels. The findings were validated using instrumental variable methods with historical data and rigorous robustness tests.

**Results:**

The construction of the SCC significantly reduced residents' medical expenditures by 10.86% (β = −0.115, *P* < 0.05). This cost-saving effect was primarily driven by optimized health behaviors and psychological wellbeing, as evidenced by increased sports consumption (β = 0.211, *P* < 0.01), higher physical activity frequency (β = 0.124, *P* < 0.05), improved self-rated health (β = 0.034, *P* < 0.1), and lower depression levels (β = −0.026, *P* < 0.1). Heterogeneity analysis indicates that residents aged 60 and above (β = −0.134, *P* < 0.05), those without chronic diseases (β = −0.098, *P* < 0.1), those without mortgage burdens (β = −0.208, *P* < 0.01), and residents living in central cities (β = −0.315, *P* < 0.01) or in areas with relatively scarce medical resources (β = −0.245, *P* < 0.1) exhibited a more pronounced reduction in medical expenditures due to SCC construction.

**Conclusion:**

This study provides compelling empirical evidence that the SCC construction effectively validates the “exercise as medicine” hypothesis at the urban governance level. By optimizing the urban health environment, the pilot project has driven a paradigm shift from passive medical treatment to proactive health management. However, the identified structural heterogeneity underscores the need for future policies to embrace inclusive precision governance. Targeted subsidies for mobility-constrained households and the integration of exercise prescriptions into chronic disease management are crucial to ensuring the equitable distribution of health dividends across all socioeconomic strata.

## Graphical Abstract

**Figure:**
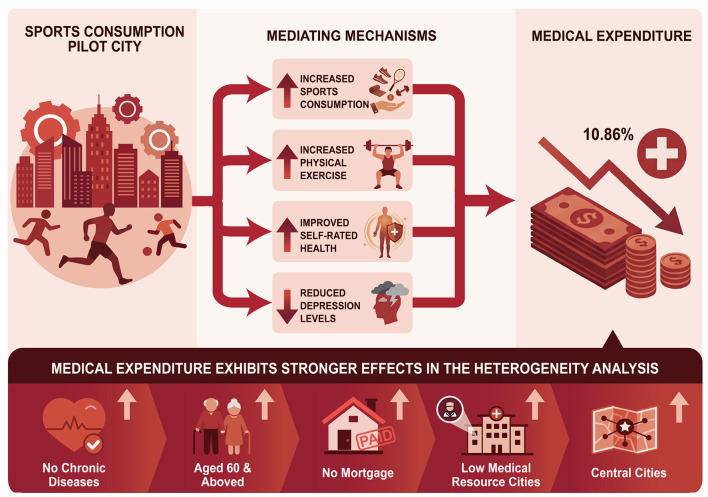

## 1 Introduction

The accelerating pace of global population aging, the persistent rise in the burden of chronic non-communicable diseases, and the continuous expansion of healthcare demand have driven rapid growth in healthcare expenditures, posing a significant practical challenge to the sustainable socioeconomic development of nations (1). A 2025 report by the Organization for Economic Cooperation and Development (OECD) indicates that healthcare expenditure as a percentage of GDP shows a pronounced trend of rigid growth in most countries. Among them, the United States, Germany, and France have maintained consistently high healthcare expenditure ratios over the long term, while South Korea, due to accelerated population aging, has become one of the economies with the fastest-growing healthcare expenditures (2). Furthermore, countries like Italy and Spain have been cautioned by the European Commission that the persistent rise in long-term care costs poses severe challenges to national fiscal sustainability (3). Existing research indicates that relying solely on expanding healthcare systems and post-event treatment interventions is insufficient to curb the rapid growth of healthcare expenditures (4). In contrast, improving residents' health behaviors, enhancing physical activity levels, and shifting the focus toward health promotion and disease prevention are considered crucial pathways for optimizing healthcare expenditure structures and transforming health governance (5). Against this backdrop, sports participation and sports consumption are increasingly being integrated into public health policy frameworks and viewed as important institutional arrangements for promoting residents' health and alleviating the burden on healthcare systems.

As one of the world's most populous developing nations, China is navigating a critical phase marked by profound demographic shifts and rapidly growing health demands among its citizens. Against this backdrop, healthcare spending has expanded rapidly. Between 2013 and 2024, per capita healthcare expenditure rose from RMB 2,316.23 to RMB 6,454.40, representing an average annual growth rate of 9.2%. This reflects sustained pressure on the healthcare security system. The underlying reasons are 2-fold: On one hand, China's population aging process has accelerated significantly. By 2020, the proportion of people aged 60 and above had reached 18.7% (6), and it is projected to exceed 28% by 2040. Simultaneously, approximately 75% of the older adults suffer from one or more chronic non-communicable diseases, substantially increasing the intensity and frequency of healthcare service utilization (7). On the other hand, constrained by developmental stages and institutional conditions, long-standing imbalances in healthcare resource allocation persist between urban and rural areas as well as across regions. The pattern of inadequate healthcare service provision in rural areas and the high concentration of quality medical resources in cities has yet to be fundamentally reversed (8). This has led to rapid growth in healthcare expenditures and health disparities among different population groups, reinforcing each other (9). How to effectively improve public health while mitigating the rigid upward pressure on healthcare costs has become a critical issue requiring urgent attention within China's public policy framework. Compared to the traditional approach of solely expanding healthcare supply, guiding residents toward more proactive and sustainable healthy lifestyles: enhancing participation in physical activity and health-related behaviors, is widely recognized as possessing greater long-term and robust policy potential.

To unlock the potential of sports consumption, improve the public sports service system, and guide residents toward active physical engagement, China continues advancing policy innovations in sports consumption. As a key initiative, the General Administration of Sport officially launched a pilot program for Sports Consumption Cities (SCC) in 2020. By selecting pilot cities to explore institutional frameworks and policy trials, the program fosters a virtuous cycle among sports consumption, urban development, and public health. The policy centers on cities as the primary spatial carriers for residents' production and daily life. It emphasizes systematically enhancing the accessibility and diversity of public sports services by improving sports facility provision and expanding sports consumption scenarios. This approach aims to stimulate residents' demand for sports consumption and promote participation in physical activities.

In terms of implementation outcomes, SCC has achieved notable successes across multiple regions, particularly by stimulating residents' demand for sports consumption and expanding the scale of sports spending. In Rizhao City, Shandong Province, the city effectively boosted sports consumption through initiatives such as promoting sports brand development, establishing outdoor fitness and leisure consumption hubs, and hosting sports-themed consumer seasons. In 2021, the city's total sports consumption reached 6.476 billion yuan, a 12.40% increase from 2020. Per capita sports consumption among residents stood at 2,179.21 yuan, up 12.27% year-on-year. Xinyu City in Jiangxi Province leveraged its industrial foundation and ecological resources to systematically advance its sports consumption pilot program. It issued the “Work Plan for Advancing the National Sports Consumption Pilot City,” outlining key tasks such as establishing a national-level sports industry base, a smart sports service platform, and fitness and rehabilitation guidance centers. Through optimizing smart sports platforms, enhancing community “15-min fitness circles,” and hosting events such as sports carnivals and sports consumption seasons, Xinyu attracted over 100,000 participants. Sports consumption vouchers worth more than 3 million yuan leveraged over 20 million yuan in direct sports spending, demonstrating a significant multiplier effect.

**Figure 1:**
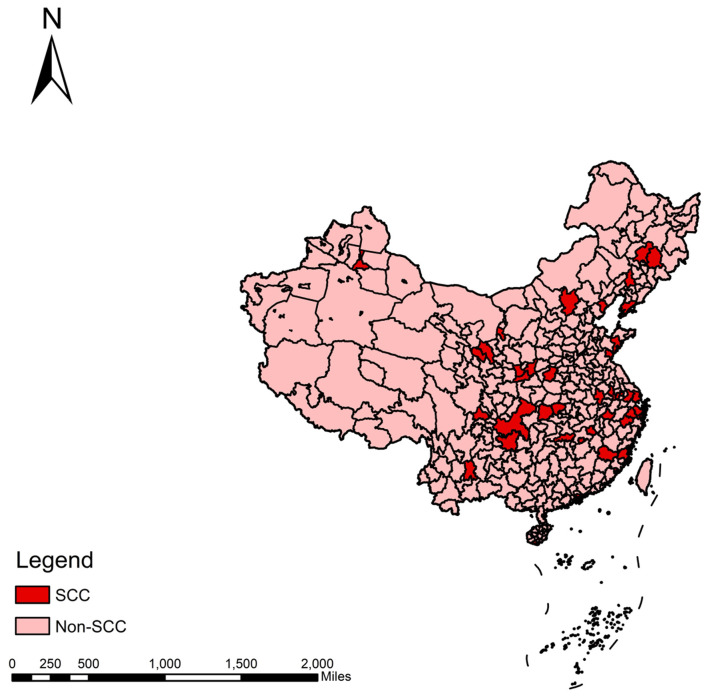
The sports consumption pilot city project.

From a macro perspective, the combined effect of a series of policy tools has led to a sustained increase in sports consumption among residents of national sports consumption pilot cities. According to data released by the General Administration of Sport of China, per capita sports expenditure among residents in the 40 SCC cities reached 2,576 yuan in 2022, a 19.65% increase from 2,153 yuan in 2020. Simultaneously, resident sports consumption surveys released by some pilot cities indicate that per capita sports spending has significantly increased in most pilot cities following policy implementation. Cities such as Shanghai and Shenzhen have approached the developed-country standard of nearly $500 per capita in sports consumption. However, while existing research fully demonstrates the positive role of SCC policy in expanding sports consumption and optimizing consumption structure, systematic micro-level empirical evidence remains lacking on whether this can further translate into substantive changes in residents' healthcare expenditures. To address this gap, this study uses CFPS micro-tracking data to construct a Difference-in-Differences (DID) model, systematically evaluating the impact of SCC construction on residents' healthcare expenditures. The treatment group comprises residents of sports consumption pilot cities, while the control group consists of residents from non-pilot cities (see Figure 1).

The potential contributions of this study are primarily reflected in three aspects. First, as a significant institutional innovation in China's sports governance, SCC construction remains in its exploratory and pilot phase. Unlike existing research that primarily focuses on the impact of sports consumption policies on sports industry development or residents' consumption structure, this study adopts a micro-individual perspective to systematically examine the policy's effect on residents' healthcare expenditures, thereby expanding the research dimensions for evaluating the effectiveness of sports consumption policies. Second, existing studies predominantly employ cross-sectional data or correlation analysis, which struggle to identify causal effects from policy implementation. This study fully leverages the quasi-natural experimental nature of SCC construction. By constructing a DID model based on CFPS micro-tracking data, it conducts causal identification of policy effects. This methodological approach effectively mitigates potential endogeneity issues, enhancing the reliability and persuasiveness of research conclusions. Finally, this study delves into the policy's operational mechanisms, examining how SCC construction may influence residents' healthcare expenditures through pathways such as increased sports consumption, increased physical activity frequency, improved self-rated health, and reduced depression levels. This provides new micro-level empirical evidence for understanding the health economics effects of sports policies.

## 2 Theoretical framework and research hypotheses

### 2.1 Direct impact of SCC construction on residents' medical expenditures

Driven by biological aging and social-environmental factors, the population's vulnerability to chronic diseases is increasing, leading to rapid growth in healthcare costs. Just as climate change exacerbates physical stress on the older adults (10), urban environments lacking health-supporting elements also amplify health risks and associated medical expenditures. Existing literature emphasizes that effective urban planning—such as optimizing public infrastructure—can mitigate health risks and promote sustainable urban development (11, 12). However, addressing the healthcare expenditure crisis cannot rely solely on expanding medical resources; it demands a systemic transformation of urban governance. The SCC initiative establishes a new paradigm for urban health governance. SCC is not merely an economic policy but a comprehensive, multidimensional upgrade of the urban service ecosystem. By optimizing the supply of public health infrastructure and reshaping a “health-friendly” urban macro-environment, SCC construction propels cities from “passive medical treatment” toward “proactive health management.” This structural optimization of the urban environment enhances cities' capacity to prevent disease at its source and sustain residents' well-being, thereby reducing the need for medical interventions and hospitalizations. Therefore, Hypothesis H1 is proposed.

H1: SCC construction can reduce residents' healthcare expenditures.

### 2.2 Indirect impact of SCC construction on residents' medical expenditures

The SCC construction aims to unlock the potential of sports consumption through institutional guidance and environmental optimization. By integrating the economic attributes and health impacts of sports consumption policies, this study constructs an analytical framework that examines the intermediary mechanisms through which SCC construction influences residents' healthcare expenditures. The framework operates on two levels: behavioral incentives and health outcomes. Based on this framework, it is argued that SCC construction does not directly affect healthcare expenditures but rather indirectly reduces them by influencing residents' health-related behaviors and perceptions of health.

From a health economics perspective, sports consumption is not merely an ordinary consumption behavior that satisfies immediate life needs. At its core, it is a “forward-looking health investment” with health-oriented attributes. Existing research indicates that higher levels of individual health investment can effectively reduce the intensity of healthcare resource utilization and generate a significant “crowding-out effect” on medical expenditures (13). For instance, every dollar invested in physical activity-focused health promotion programs can reduce healthcare costs by approximately $3.27 (14). Furthermore, household expenditures on sports and health-related products and services exhibit a significant negative correlation with subsequent healthcare spending—higher levels of sports-related consumption are associated with lower healthcare expenditure burdens for individuals or households (15). Through policy tools such as sports consumption subsidies and the creation of diverse consumption scenarios, SCC construction effectively lowers the economic costs and institutional barriers to sports participation, thereby increasing residents' sports consumption. This policy-driven optimization of the consumption structure encourages residents to allocate more resources toward front-end health prevention rather than back-end disease treatment, curbing healthcare expenditure growth at its source. Therefore, we propose Hypothesis H2.

H2: SCC construction reduces healthcare expenditures by increasing residents' sports consumption.

In residents' daily lives, the continuity of physical activity often depends on material and service inputs, including sports equipment, functional apparel, and various fitness and sports services. The SCC construction has reduced constraints on residents' participation in sports activities at both the institutional and cost levels by enhancing diverse sports participation scenarios, thereby improving the external environment and accessibility of physical exercise. This policy arrangement not only provides practical support for physical activity but also facilitates the transition of residents from occasional to regular participation. As the frequency and consistency of physical activity increase, residents' overall health risks tend to decrease, reducing their reliance on healthcare services and, consequently, lowering medical expenditures (16). Therefore, we propose Hypothesis H3.

H3: SCC construction reduces medical expenditures by increasing residents' frequency of physical activity.

Residents' health status is a core determinant of healthcare utilization and expenditure levels. Self-rated health provides a relatively comprehensive reflection of an individual's physical functioning and overall health perception (17), exhibiting significant correlations with healthcare utilization behavior and medical expenditure (18, 19). Generally, higher self-rated health levels indicate lower susceptibility to illness and health risks, leading to reduced dependence on healthcare services and consequently lower healthcare expenditures (20). The implementation of the SCC program creates a more health-conducive external environment by optimizing public sports service provision and enhancing access to sports resources. Against this backdrop, residents' overall health perception improves, leading to enhanced self-rated health status. The improvement in self-rated health not only reflects a reduction in individual health risks but also decreases the frequency and intensity of healthcare service demand at the behavioral level, thereby exerting a restraining effect on healthcare expenditures. Therefore, Hypothesis H4 is proposed.

H4: SCC construction reduces healthcare expenditures by improving residents' self-rated health levels.

Mental health status is a significant non-physiological factor influencing healthcare utilization and expenditure levels (21). Existing research indicates that lower depression levels can effectively reduce the frequency of healthcare service use, thereby lowering the burden of medical expenses (22). By strengthening sports consumption orientation and optimizing the consumption environment, SCC construction comprehensively improves residents' psychological wellbeing and life experiences, thereby influencing their depression levels. As depression decreases, residents' reliance on healthcare services correspondingly declines, leading to reduced medical expenditures. Therefore, we propose Hypothesis H5:

H5: SCC construction reduces medical expenditures by reducing residents' depression levels.

## 3 Study design and methods

### 3.1 Data source

The data used in this study were drawn from the China Family Panel Studies (CFPS) and the China Urban Statistical Yearbook. CFPS is a nationwide, long-term longitudinal survey project organized and implemented by the Center for Social Science Surveys at Peking University. It systematically collects information on socioeconomic characteristics, health status, and behavioral patterns at both the household and individual levels, demonstrating high representativeness and reliability. We acknowledge that CFPS data may introduce some degree of sample selection bias due to unavoidable attrition from causes such as death, migration, or refusal to participate. The five waves of CFPS surveys from 2014 to 2022 cover the implementation period of the SCC and are sufficient for our research needs. After data cleaning, the final sample included 6,484 individuals.

### 3.2 Empirical model construction and variables selection

The SCC construction initiative provides a typical quasi-natural experimental setting for this study. The policy was implemented in 2020 through a single centralized selection and pilot program, with all pilot cities completing their cycles by 2022. It did not exhibit characteristics of phased rollouts or continuous expansion. Consequently, the policy shock has clear and consistent implementation timelines, making it suitable for DID analysis to assess the impact of SCC construction on residents' healthcare expenditures. The DID model for this study is specified as follows:


$$\begin{array}{l} Y_{ict}=\beta_{0}+\beta_{1}Treat_{ic}\times Time_{t}+\beta_{2}X_{ict}+\beta_{3}Z_{ct}+\mu_{i}+\theta_{t}\\ \;+\varepsilon_{it} \end{array}$$ (1)

In equation 1, *Y*_*ict*_ denotes the healthcare expenditure of resident *i* in city *c* during year *t*. *Treat*_*ic*×_*Time*_*t*_ serves as the core explanatory variable. β_1_ represents the policy effect of SCC. *X*_*ict*_ represents a set of individual and household-level control variables, while *Z*_*ct*_ represents a set of city-level macro control variables. β_2_ and β_3_ denote their corresponding regression coefficients. β_0_ is the intercept term, μ_*i*_ is the individual fixed effect, and θ_*t*_ is the time fixed effect. ε_*it*_ is the random disturbance term.

The explained variable in this study is Residents' Medical Expenditures (RME). Constructed from CFPS data, this variable primarily aggregates an individual's total hospitalization costs with other illness- or injury-related medical expenditures, serving as a comprehensive indicator of actual healthcare service utilization. Given the pronounced right-skewed distribution of the medical expenditure variable and the presence of zero values in some samples, this study adds one to each raw medical expenditure value before taking the natural logarithm. This transformation reduces the impact of extreme values and enhances the robustness of regression results. Higher values of this indicator indicate greater levels of individual medical expenditure.

The core explanatory variable in this study is SCC construction. By integrating CFPS data with the SCC construction list, this study constructs an SCC policy dummy variable to identify the impact of SCC construction on medical expenditures. To align with the timeline of the policy shock, this study focuses on examining the effectiveness of SCC implementation utilizing five waves of the CFPS data collected in 2014, 2016, 2018, 2020, and 2022.

First, this study enhances sports consumption. Sports consumption is measured using questions on household health and wellness expenditures in the CFPS questionnaire. The CFPS captures actual household spending on sports, fitness, and related health domains through the question: “How much does your household spend on health-related expenses for fitness activities, purchasing related products and equipment, health supplements, etc.?” This indicator encompasses expenditures on fitness activities and on products and equipment directly related to sports activities, providing a relatively comprehensive reflection of residents' investment levels in the sports and health sectors. Because this variable is measured at the household level while this study focuses on the individual level, household health care expenditures are adjusted per capita based on household size. This yields a per capita household sports consumption expenditure indicator that characterizes the average level of resources available for sports and health investments within each individual's household. In the empirical analysis, this variable undergoes a +1 ln transformation.

Second, this study increases the frequency of physical activity. Physical activity frequency is measured using the “frequency of participating in sports, fitness, and recreational activities” question from the CFPS questionnaire. This question explicitly excludes walking or cycling solely for commuting and encompasses all types of sports, fitness, and recreational activities, including physical education classes. It provides a relatively comprehensive reflection of residents' actual participation in physical activity. This indicator is measured using an ordered categorical variable ranging from 1 to 8. Values increase sequentially from “never participate” to “twice or more daily,” with higher numbers indicating greater levels of individual physical activity participation.

Third, this study improves self-rated health. Self-rated health was measured using the question “How would you rate your current health?” from the CFPS questionnaire (23). This indicator was constructed using a five-point Likert scale, with respondents assigning scores from 1 to 5 based on their subjective evaluation of their health, ranging from low to high. These scores correspond to “poor,” “fair,” “good,” “very good,” and “excellent,” respectively. A higher score indicates a higher level of self-rated health.

Fourth, this study reduces depression levels. This variable was measured using the Center for Epidemiologic Studies Depression Scale (CES-D-8) from the CFPS annual survey, the standard tool used in CFPS to evaluate depressive symptoms. The CES-D-8 comprises eight items that assess the frequency of relevant emotions and behaviors experienced by respondents over the past week. Each item is scored on a four-point scale from 1 to 4, with two positive-affect items (feeling happy, feeling cheerful) reverse-scored. This yields a depression score ranging from 8 to 32 points, with higher scores indicating greater depression severity (24).

### 3.3 Statistical description

To control for the influence of other variables, the following control variables were selected: at the individual level, age, gender, marital status, and education level; at the household level, household size, number of older adults; at the city level, per-capita GDP, number of hospital beds, local fiscal health spending. All variable definitions are presented in Table 1.

**Table 1:** Descriptive statistics of variables.

**Type**	**Variable**	**Definition**	**Mean**	**SD**	**Min**	**Max**
Explained variables	RME	Ln (residents' medical expenditure+1)	4.925	3.613	0.000	12.900
Core explanatory variables	SCC	Sports consumption cities construction	0.047	0.212	0.000	1.000
Mediator variables	Sports consumption	Ln (personal sports consumption+1)	1.653	3.078	0.000	11.982
	Physical activity	Frequency of participation in physical activities	5.059	2.055	1.000	8.000
	Self-rated health	Self-rated health score	2.903	1.144	1.000	5.000
	Depression	CES-D-8 score	13.221	3.840	8.000	32.000
Control variables	Age	Age of respondent	53.170	13.100	16.000	89.000
	Gender	Gender of respondent	0.554	0.497	0.000	1.000
	Marry	Marital status of respondent	8.762	4.655	0.000	19.000
	Edu	Years of education of respondents	2.378	1.453	0.000	9.000
	Family	Number of family members	3.731	1.834	1.000	15.000
	Elder	Number of older adults	0.573	0.790	0.000	4.000
	Per-capita GDP	Ln (per-capita GDP)	10.899	0.440	10.131	12.154
	Number of hospital beds	Ln (number of hospital beds)	10.185	0.861	7.912	12.134
	Local fiscal health spending	Ln (local fiscal health spending)	13.150	1.020	11.047	16.387

For dummy variables SCC, the mean represents the proportion of observations with a value of 1.

## 4 Results and analysis

### 4.1 Baseline regression results

Table 2 reports the impact of SCC construction on RME. Model (1) presents the results of the baseline regression without control variables, while Model (2) reports the results after introducing control variables. In both Models (1) and (2), SCC has a significant negative effect on RME, with coefficients ranging from −0.197 to −0.115. After controlling for various factors, SCC reduces household medical expenditures by 10.86% (e^−0.115^-1).

**Table 2:** Medical expenditure effects of SCC pilots.

Variables	(1)	(2)
	RME	RME
SCC	−0.197^**^	−0.115^**^
	(0.096)	(0.058)
Covariates	NO	YES
R^2^	0.617	0.621
IFE	YES	YES
TFE	YES	YES

Robust standard errors clustered at the city level are reported in parentheses. ^*^ means *P* < 0.1, ^**^ means *P* < 0.05, ^***^ means *P* < 0.01.

### 4.2 Model validity testing

**Figure 2:**
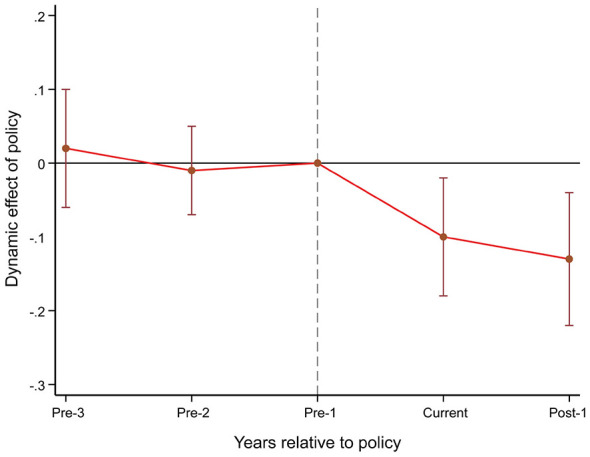
Parallel trends test for residents' medical expenditure.

The parallel trends assumption is a crucial prerequisite for evaluating policy effects using DID methods, meaning that prior to SCC implementation, the trends in healthcare expenditure changes among residents in the treatment and control groups should remain consistent. To test this assumption, the event study method was employed to estimate the policy's dynamic effects and to systematically examine parallel trends. Specifically, the interaction term between the policy implementation dummy variable and the year dummy variable was included in the regression model, with 2018 (two years before policy implementation) serving as the baseline reference period. The results in Figure 2 indicate that prior to 2018, the difference in RME between pilot and non-pilot cities was not significant, thereby satisfying the parallel trends assumption.

### 4.3 Placebo test

**Figure 3:**
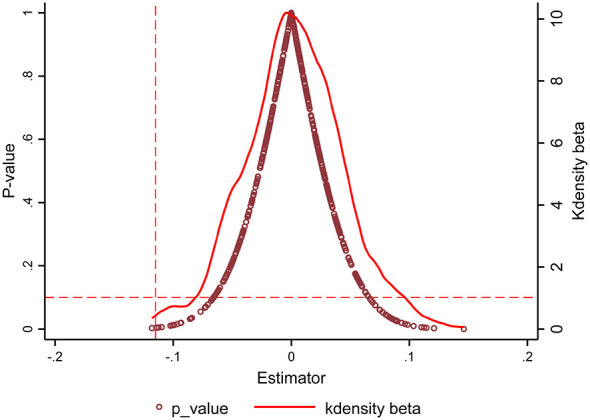
Placebo test for residents' medical expenditure.

To further test the robustness of the benchmark regression results and prevent estimates from being influenced by random factors or unobservable shocks, a placebo test was conducted to assess the policy effects of SCC by randomly designating pilot cities (25). This study randomly selected 40 cities from the full sample, virtually designated them as SCC cities, and constructed a “pseudo” policy treatment variable accordingly. Under unchanged model specifications, the impact of SCC on RME was re-estimated. Using Monte Carlo simulation, the random assignment process was repeated 500 times to obtain 500 “pseudo” policy regression coefficients and their corresponding *p*-values, whose distribution is shown in Figure 3. The results indicate that most pseudo-regression coefficients are statistically insignificant and deviate markedly from the estimated results of the true policy shock in the benchmark regression. This suggests that the policy effect of SCC in the benchmark regression is not attributable to random assignment but rather represents a low-probability event in the tail of the placebo test distribution.

### 4.4 Endogenous treatment

To address potential endogeneity issues in estimating the policy effects of the SCC, we employ two-stage least squares (2SLS) and introduce historical instrumental variables. Specifically, we use the number of individuals from each region who passed the Ministry of War's military examinations (“military jinshi”) during the Ming Dynasty, as recorded in the Ming Dynasty Military Examination Database, as instrumental variables.

The relevance of this instrumental variable stems from the rigorous physical fitness and martial arts requirements of the ancient military examination system. A higher number of military jinshi indicates that a region possesses a stronger historical foundation in sports culture and martial traditions. Through long-term path dependence mechanisms, such deep-rooted cultural characteristics shape the contemporary local sports development environment and infrastructure needs, thereby significantly influencing the likelihood of a city being selected as a “National Sports Consumption Pilot City.”

However, a potential concern is that the large-scale wars and mass population migrations accompanying the transition from the Ming to the Qing dynasty (such as the “Huguang Fills Sichuan” migration documented in historical records) may have weakened or even severed the biological and intergenerational continuity of local cultural characteristics. To address this issue of historical endogeneity, we adopted a strategy combining theoretical and empirical approaches.

First, at the theoretical level, the literature on historical economics indicates that deeply entrenched cultural and social norms often exhibit strong spatial continuity. As long as the local natural environment and survival incentives remain stable, the benefits of intergenerational transmission of specific social norms are maximized (26). Therefore, even in the face of major historical demographic shocks such as the “Huguang Fills Sichuan” migration, new immigrants typically undergo “spatial assimilation” to adapt to the environment, as the region's unique geographical endowments or strategic defense needs remain unchanged (27). During this assimilation process, the new immigrants inherited and perpetuated the region's existing martial arts and sports traditions. This also corroborates the view in cultural evolution theory that large-scale population movements do not completely erase the inherent cultural characteristics of different regions, and that the geographical environment exerts a long-term path dependence on cultural formation (28, 29).

Second, on the empirical level, we not only report the results of instrumental variable regressions for the full sample but also conduct rigorous robustness tests: we exclude regions that have historically experienced extreme population turnover or social upheaval (including Sichuan Province and Chongqing Municipality, which were affected by large-scale migration, as well as Jiangsu, Zhejiang, and Anhui provinces, which suffered severe population losses during the Taiping Rebellion).

The empirical results are shown in Table 3. In the baseline regression of the full sample, the results of the first stage of Model (3) indicate a significant positive correlation between the instrumental variable and the SCC variable (β = 0.358, *P* < 0.01). The Kleibergen-Paap Wald F-statistic reaches 31.457, far exceeding the empirical threshold of 10, thereby strongly ruling out concerns regarding weak instrumentality; The results of the second stage of Model (4) show that SCC still has a significant negative impact on household medical expenditures (β = −0.197, *P* < 0.1). More importantly, in robustness tests that exclude specific regions affected by historical turmoil (Models 5 and 6), even with a reduced sample size, the F-statistic in the first stage remains as high as 25.544. The second-stage estimation results of Model (6) indicate that, after strictly controlling for endogeneity and historical population shocks, the negative impact of SCC on healthcare expenditure remains robust and significant (β = −0.051, *P* < 0.05). This not only fully aligns with the conclusions of our revised baseline study but also further confirms the validity of the “spatial assimilation” theory—namely, that within the broader macro-spatial context of China, the deep-rooted tradition of sports culture has not been disrupted by localized population shocks.

**Table 3:** Results of instrumental variable approach (2SLS).

Variables	First stage	Second stage	First stage	Second stage
	(3)	(4)	(5)	(6)
	SCC	RME	SCC	RME
SCC		−0.197^*^		−0.051^**^
		(0.117)		(0.022)
IV	0.358^***^		0.135^***^	
	(0.124)		(0.046)	
Exclude specific historical regions	NO	NO	YES	YES
Covariates	YES	YES	YES	YES
Kleibergen-Paap LM	38.851^***^		24.367^***^	
Kleibergen-Paap Wald F	31.457^***^		25.544^***^	
R^2^	0.773	0.781	0.773	0.781

Robust standard errors clustered at the city level are reported in parentheses.

^*^ means *P* < 0.1,

^**^ means *P* < 0.05,

^***^ means *P* < 0.01.

### 4.5 Robustness check

#### 4.5.1 Tail trimming of dependent variable

Given that RME is susceptible to individual health shocks and sporadic medical events, its distribution may include extreme values. To mitigate the potential influence of outliers on regression results, the original RME variable underwent 5% two-tailed trimming. Based on this, RME was reconstituted as the dependent variable for regression analysis. The results of Model (7) in Table 4 show that the estimated coefficient for SCC is negative (β = −0.098, *P* < 0.1), consistent with the benchmark regression results and further validating the robustness of the study's conclusions.

**Table 4:** Robustness test results.

Variables	(7)	(8)	(9)	(10)	(11)	(12)
	RME	RME	RME	RME	RME	RME
SCC	−0.098^*^	−0.126^**^	−0.113^**^	−0.128^*^	−0.077^***^	−0.111^**^
	(0.057)	(0.055)	(0.049)	(0.071)	(0.028)	(0.050)
IMNC		−0.098^*^		−0.121^**^		
		(0.053)		(0.058)		
HC			−0.124^***^	−0.223^***^		
			(0.042)	(0.075)		
Covariates	YES	YES	YES	YES	YES	YES
Covariates^2^	NO	NO	NO	NO	NO	YES
R^2^	0.653	0.644	0.445	0.624		
IFE	YES	YES	YES	YES	YES	YES
TFE	YES	YES	YES	YES	YES	YES

Robust standard errors clustered at the city level are reported in parentheses.

^*^ means *P* < 0.1,

^**^ means *P* < 0.05,

^***^ means *P* < 0.01.

#### 4.5.2 Exclusion of other policy interferences

During the implementation of the SCC pilot program, other pilot programs, such as Integrated Medical and Nursing Care (IMNC) (30) and Healthy Cities (HC) (31), were also carried out concurrently. These pilot programs share similarities in improving public health environments and enhancing access to health services. To mitigate the confounding effects of concurrent policy implementation, this study further incorporated dummy variables for IMNC and HC as control variables in the baseline regression model [Equation 1], thereby isolating the influence of contemporaneous policies. The results of Models (8), (9), and (10) in Table 4 indicate that after excluding IMNC (β = −0.126, *P* < 0.05), HC (β = −0.113, *P* < 0.05), and both IMNC and HC (β = −0.128, *P* < 0.1), SCC construction still reduces RME, thereby supporting the reliability of the findings.

#### 4.5.3 Replacement of estimation method

To further mitigate the potential impact of model specification bias and functional form assumptions on estimation results, this study introduces a double machine learning approach and employs the random forest algorithm to re-estimate and predict the benchmark regression (32). The results are presented in Table 4. Model (11) includes the first-order linear term of the control variable (β = −0.077, *P* < 0.01). Model (12) further includes the quadratic term of the control variable (β = −0.111, *P* < 0.05). Both coefficients are negative, suggesting that our core conclusions are robust.

#### 4.5.4 Handling the pandemic

The COVID-19 pandemic presents a significant potential confounding factor in accurately estimating the true effect of the SCC policy. A critical identification challenge is distinguishing whether the observed reduction in residents' medical expenditures is driven by genuine “health improvements” resulting from sports consumption, or merely a statistical artifact of “lockdown-induced delayed medical care” (i.e., residents being restricted from visiting hospitals during outbreaks). To rigorously isolate the pure policy effect from the pandemic's interference, we conduct robustness checks from both temporal and spatial dimensions.

First, from a temporal perspective, we completely exclude the sample from the year 2022, a period characterized by widespread outbreaks and strict lockdown measures in various Chinese cities. As reported in Model (13) of Table 5, after excluding this major exogenous shock, the coefficient of SCC remains significantly negative (β = −0.085, *P* < 0.05).

**Table 5:** Handling the pandemic.

Variables	(13)	(14)
	RME	RME
SCC	−0.085^**^	−0.046^*^
	(0.039)	(0.024)
Number of confirmed cases	NO	YES
Mobility indicators	NO	YES
Covariates	YES	YES
R^2^	0.689	0.731
IFE	YES	YES
TFE	YES	YES

Robust standard errors clustered at the city level are reported in parentheses.

^*^ means *P* < 0.1,

^**^ means *P* < 0.05.

Second, from a spatial and intensity perspective, we re-incorporate the 2022 sample but explicitly control for the heterogeneity of the pandemic's severity and the strictness of movement restrictions across different cities. Specifically, we integrate two key city-level control variables into the regression model:

The severity of the outbreak, measured by the number of confirmed COVID-19 cases sourced from the local Health Commissions.

Urban mobility restrictions, proxied by the Gaode Map Congestion Delay Index. This index, derived from massive vehicle trajectory data, calculates the ratio of actual travel time to free-flow travel time. During the pandemic, a lower congestion index accurately reflects a significant reduction in urban traffic and the strict enforcement of stay-at-home orders.

As shown in Model (14), even after strictly absorbing the “medical avoidance” noise caused by local infection risks and traffic lockdowns, the SCC continues to exert a significant negative impact on medical expenditures (β = −0.046, *P* < 0.1). Together, these dual-dimensional tests firmly establish that the decline in medical expenditures is a genuine, policy-driven health dividend.

#### 4.5.5 Replacement of the matching method

**Figure 4:**
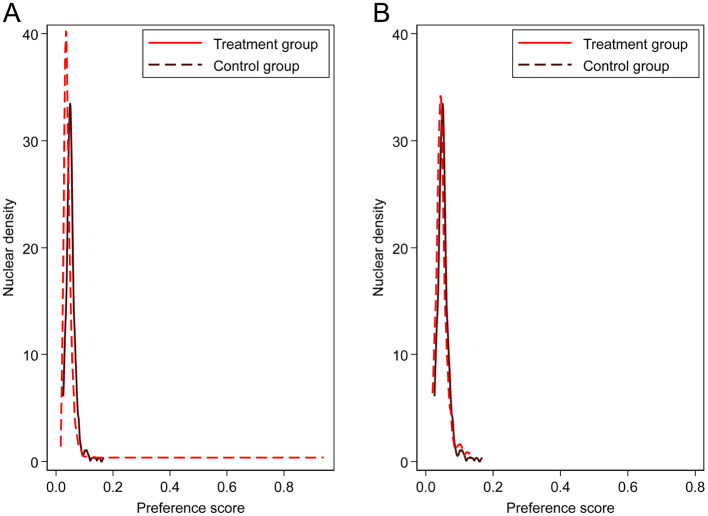
Propensity score kernel density distribution. **(A)** represents pre-psm, **(B)** represents post-psm.

To mitigate the potential impact of sample selection bias on estimation results, we employed the PSM-DID method to conduct additional robustness tests. Using the individual- and household-level control variables from the baseline regression, we first performed 1:1 nearest-neighbor matching between pilot and non-pilot city samples. Figure 4 shows the propensity score kernel density distributions for the treatment and control groups before and after matching. The results indicate high overlap between the two groups post-matching, suggesting satisfactory matching quality. Based on this, we re-estimated the DID model using the matched samples.

Furthermore, to assess the sensitivity of matching results to different strategies, this study re-matched the samples using both kernel and radius matching (with a caliper set to 0.01) and repeated the DID estimation on the re-matched samples. Table 6 shows that whether using 1:1 Nearest Neighbor Matching (β = −0.042, *P* < 0.01), Kernel Matching (β = −0.194, *P* < 0.1), or Radius Matching (calipers set to 0.01) (β = −0.072, *P* < 0.1), the SCC-PSM coefficients were largely consistent with the baseline regression, further validating the robustness of the conclusions presented in this study.

**Table 6:** Replacement of the matching method.

Variables	1:1 Nearest neighbor matching	Kernel matching	Radius matching (Calipers set to 0.01)
	(15)	(16)	(17)
	RME	RME	RME
SCC	−0.042^***^	−0.194^*^	−0.072^*^
	(0.014)	(0.101)	(0.043)
Covariates	YES	YES	YES
R^2^	0.699	0.584	0.617
IFE	YES	YES	YES
TFE	YES	YES	YES

Robust standard errors clustered at the city level are reported in parentheses.

^*^ means *P* < 0.1,

^***^ means *P* < 0.01.

### 4.6 Mechanism of action analysis

To uncover the mechanism by which SCC construction influences RME, a two-step approach is used to examine the mediating effects of intermediate variables between the explanatory and outcome variables. This two-step method helps mitigate potential endogeneity issues to some extent. This section will systematically test the relevant mediating pathways to validate the research hypotheses H2–H5 proposed earlier.

We selected sports consumption, physical activity frequency, self-rated health, and depression levels as mediating variables. The regression results are shown in Table 7. Model (18), with sports consumption as the explained variable, yielded a positive SCC coefficient (β = 0.211, *P* < 0.01). This indicates that SCC construction can increase residents' sports consumption. Higher sports consumption encourages residents to allocate more resources toward health-enhancing lifestyle investments, thereby improving individual health capital accumulation and reducing future healthcare service utilization, ultimately curbing medical expenditures. Model (19) uses physical activity frequency as the explained variable, with a positive SCC coefficient (β = 0.124, *P* < 0.05). This indicates that SCC construction can increase the frequency of residents' participation in physical activity. Higher physical activity frequency improves individual physical fitness, thereby reducing disease risks and healthcare needs. Model (20) uses self-rated health as the explained variable, with a positive SCC coefficient (β = 0.034, *P* < 0.1). This demonstrates that SCC construction can improve residents' subjective health perceptions and improves their overall health evaluations. Model (21) uses depression levels as the explained variable, with a negative SCC coefficient (β = −0.026, *P* < 0.1). This indicates that SCC construction reduces residents' depression levels. Alleviating negative emotions and psychological stress contributes to improving the overall health status of middle-aged and older adults.

**Table 7:** Results of mechanism of action analysis.

Variables	(18)	(19)	(20)	(21)
	Sports consumption	Physical activity	Self-rated health	Depression
SCC	0.211^***^	0.124^**^	0.034^*^	−0.026^*^
	(0.073)	(0.055)	(0.018)	(0.015)
Covariates	YES	YES	YES	YES
R^2^	0.666	0.639	0.628	0.634
IFE	YES	YES	YES	YES
TFE	YES	YES	YES	YES

Robust standard errors clustered at the city level are reported in parentheses.

^*^ means *P* < 0.1,

^**^ means *P* < 0.05,

^***^ means *P* < 0.01.

### 4.7 Heterogeneity analysis

Individual differences and uneven urban development conditions may lead to variations in the effectiveness of SCC construction. Therefore, heterogeneity analysis is conducted at both the micro-individual and macro-urban levels.

#### 4.7.1 Micro-level analysis of individual heterogeneity

At the micro-individual level, the sample was grouped for regression analysis based on whether they had chronic diseases (assigned a value of 1 if present, 0 if absent), age structure (age < 60 and age ≥ 60), and whether they had a mortgage (assigned a value of 1 if present, 0 if absent).

Results in Table 8 for models (22) and (23) indicate that SCC construction has a more positive effect on individuals without chronic conditions (β = −0.098, *P* < 0.1). This may reflect that individuals without chronic conditions typically have better initial health status and greater health behavior plasticity. Consequently, this group is more likely to increase health investments and exercise, thereby effectively preventing disease onset and reducing healthcare service demand. In contrast, individuals with chronic diseases exhibit greater rigidity in their healthcare expenditures, making it difficult to significantly alter their existing healthcare demand structure in the short term.

**Table 8:** Results of micro-level individual heterogeneity analysis.

Variables	(22)	(23)	(24)	(25)	(26)	(27)
	Chronic	Non-chronic	Age<60	Age ≥60	Mortgage	No-mortgage
SCC	−0.074	−0.098^*^	−0.209	−0.134^**^	−0.129	−0.208^***^
	(0.053)	(0.057)	(0.231)	(0.066)	(0.112)	(0.064)
Covariates	YES	YES	YES	YES	YES	YES
R^2^	0.629	0.641	0.638	0.633	0.413	0.506
IFE	YES	YES	YES	YES	YES	YES
TFE	YES	YES	YES	YES	YES	YES

Robust standard errors clustered at the city level are reported in parentheses.

^*^ means *P* < 0.1,

^**^ means *P* < 0.05,

^***^ means *P* < 0.01.

Results in Table 8 for Models (24) and (25) indicate that individuals aged 60 and older (β = −0.134, *P* < 0.05) are more sensitive to health risks, bear a relatively heavier burden of medical expenditures, and show stronger marginal responses to public health interventions and health promotion policies. SCC construction, by enhancing access to age-friendly sports services and creating urban environments conducive to daily physical activity, more readily encourages older adults to increase regular exercise. This approach thus plays a more pronounced role in disease prevention and health maintenance.

Housing, as the most significant asset and source of wealth for most households, holds particular economic and social significance in Chinese society (33). Homeownership facilitates long-term wealth accumulation, expands social networks, and enhances social capital, thereby improving economic standing and social mobility. Conversely, households burdened by housing loans often face greater long-term debt repayment pressures and liquidity constraints, making their consumption patterns and health investment decisions more susceptible to financial constraints. The results of Models (26) and (27) in Table 8 indicate that the impact of SCC construction is more pronounced among mortgage-free individuals (β = −0.208, *P* < 0.01). This may stem from their relatively lower financial pressure, which facilitates SCC adoption. Conversely, mortgage-burdened individuals face stronger rigid constraints on housing expenditures, limiting their capacity to adjust health behaviors and thereby constraining the effectiveness of SCC implementation.

#### 4.7.2 Macro-level analysis of urban heterogeneity

At the macro-urban level, we further examine variation in SCC construction outcomes from the perspectives of medical resource endowment and urban functional structure. Urban healthcare resource endowment is measured by the number of licensed (assistant) physicians reported in each city's statistical yearbook. Sample cities are grouped by the median physician count over the sample period: cities with physician counts above the median are classified as high-resource, while those below the median are classified as low-resource. Urban functions are categorized as central vs. non-central cities according to the State Council's approval of the National Territorial Space Master Plan.

Results in Table 9 for Models (28) and (29) indicate that SCC construction significantly suppresses medical expenditures among urban residents in cities with relatively scarce medical resources (β = −0.245, *P* < 0.1). From a healthcare resource allocation perspective, this may reflect residents in resource-constrained settings adopting alternative pathways to ease healthcare expenditure pressures. Specifically, they increase sports-related consumption and improve health status to reduce the risk of disease incidence. Conversely, in cities with more abundant healthcare resources, residents can more readily access medical services to improve health, thereby diminishing the marginal impact of the policy effect.

**Table 9:** Results of macro-level urban heterogeneity analysis.

Variables	(28)	(29)	(30)	(31)
	Low medical resource city	High medical resource city	Central city	Non-central city
SCC	−0.245^*^	−0.138	−0.315^***^	0.117
	(0.131)	(0.097)	(0.118)	(0.084)
Covariates	YES	YES	YES	YES
R^2^	0.637	0.701	0.651	0.624
IFE	YES	YES	YES	YES
TFE	YES	YES	YES	YES

Robust standard errors clustered at the city level are reported in parentheses.

^*^ means *P* < 0.1,

^***^ means *P* < 0.01.

Significant disparities exist between central and non-central cities in infrastructure levels, public service provision, and resource allocation capabilities, which may lead to different impact pathways for SCC construction outcomes. Results from Models (30) and (31) in Table 8 indicate that SCC construction has a more pronounced effect on residents in the central city group (β = −0.315, *P* < 0.01). This may stem from central cities' relative advantages in sports consumption supply systems, marketization levels, and policy implementation capabilities. These strengths enable policy incentives to translate more effectively into actual sports consumption behaviors among residents, further reducing healthcare expenditures through health improvement mechanisms. In contrast, non-central cities face constraints due to insufficient sports consumption supply and inadequate consumption environments, limiting the release of policy effects.

From the results of the macro-level urban heterogeneity analysis, the RME-suppressing effect of SCC construction exhibits dual heterogeneity: “demand-constrained” and “supply-advantaged.” On the one hand, in cities with relatively scarce medical resources, sports consumption more effectively alleviates RME pressure through health substitution effects. On the other hand, in central cities, a well-developed sports consumption environment and strong policy implementation capacity further amplify the policy effect. This indicates that the effectiveness of SCC construction depends on residents' health demand structures and is constrained by urban hierarchy and foundational consumption conditions.

## 5 Discussion and conclusion

### 5.1 Discussion

The SCC construction is regarded as a pioneering strategic initiative by China to align the growth of its sports industry with public health objectives. Against the backdrop of global healthcare systems grappling with the rigid rise in medical expenditures driven by population aging, this study employs a rigorous DID method to evaluate the SCC program. We provide compelling evidence that this city-level non-medical intervention is an effective cost-control strategy, adding a new dimension to empirical research in health economics.

Research findings indicate that the SCC construction significantly suppressed RME growth, achieving a 10.86% reduction (β = −0.115, *P* < 0.05) in pilot cities compared with non-pilot regions. This outcome stands in stark contrast to the typical upward trend in healthcare costs observed after traditional supply-side reforms, such as hospital expansion (34). Instead, it strongly corroborates Grossman's “health capital theory” (13), which posits that increased proactive health investments can effectively “crowd out” demand for reactive medical treatments. By reducing the need for healthcare services at their source, the SCC construction supports the hypothesis that “optimizing urban health environments constitutes a fiscally sustainable strategy.”

Unlike previous studies that focused on single physiological outcomes, our mechanistic analysis reveals that this cost-saving effect is driven by four distinct, parallel pathways spanning behavioral and psychological dimensions: First, SCC construction stimulated sports consumption (β = 0.211, *P* < 0.01). Consistent with Humphreys et al. (15), we found that as households allocated more budget to sports goods and services, their reliance on healthcare services decreased, reflecting a substitution effect between “health maintenance expenditures” and “disease treatment expenditures.” Second, increased physical activity frequency (β = 0.124, *P* < 0.05) directly enhances physiological resilience. This aligns with the consensus that “exercise is medicine” (35), indicating that regular activity slows the natural depreciation of health capital. Third, improved self-rated health (β = 0.034, *P* < 0.1) serves as a subjective barrier to unnecessary medical visits. Residents with higher self-efficacy are less likely to consume healthcare resources for minor ailments (19). Fourth, the program reduced depression levels (β = −0.026, *P* < 0.1). This finding extends recent literature linking psychological distress to physical symptom reporting (22). By alleviating mental burdens, SCC programs decrease psychosomatic hospitalizations, acting as an “invisible stabilizer” for healthcare costs.

Heterogeneity analysis reveals structural limits to policy effectiveness, offering insights that both challenge and support existing theories.

At the micro level, we observe that the suppressive effect of healthcare expenditures is more pronounced among adults aged 60 and older (β = −0.134, *P* < 0.05). This finding challenges earlier assumptions that lifestyle interventions yield diminishing returns among older adults due to ingrained habits (36). Instead, it suggests that older adults—facing accelerated health capital depreciation—derive greater marginal utility from accessible sports infrastructure than younger cohorts (37). Notably, this effect is significant among individuals without chronic diseases (β = −0.098, *P* < 0.1) but diminishes among those with prior illnesses. This suggests that SCC construction primarily functions as a “primary prevention” tool targeting healthy populations rather than as therapeutic interventions. Healthcare expenditures for individuals with chronic diseases typically exhibit “demand inelasticity,” making them less responsive to lifestyle interventions in the short term (38). Consistent with “liquidity constraints” (39), residents without mortgage debt experienced a more pronounced reduction in healthcare costs (β = −0.208, *P* < 0.01). This indicates that financial liquidity is a prerequisite for participating in sports consumption. Households burdened by high debt may perceive sports participation as a luxury, thereby limiting their response to policy incentives and missing out on health dividends. From a macro perspective, the policy exhibits dual logic. In cities with scarce healthcare resources (β = −0.245, *P* < 0.1), it triggers a “substitution effect,” where residents substitute scarce medical services with sports and fitness activities. In central cities (β = −0.315, *P* < 0.01), it relies on “supply-side advantages,” where a mature sports market facilitates rapid behavioral shifts.

However, several key limitations warrant attention and point to directions for future research. First, regarding data accuracy, our analysis relies on self-reported data from the CFPS. While the survey's rigorous stratified random sampling design mitigates systematic selection bias, measurement error from recall bias remains. This implies readers should exercise caution when interpreting specific magnitude estimates, though it does not undermine the robustness of the trend direction.

Second, regarding the temporal context, our observation window inevitably overlapped with the COVID-19 pandemic. Although we employed rigorous dual-dimensional robustness checks—including the exclusion of anomalous outbreak years and the explicit control for city-level infection severity and mobility restrictions to effectively absorb short-term “medical avoidance” noise—observational data inherently limits the complete elimination of all unobservable, long-term psychological or structural shifts caused by the macro-shock. Consequently, whether the health-promoting behaviors and sports consumption habits fostered by the SCC policy will persistently translate into long-term dividends in a fully normalized post-pandemic era requires future longitudinal validation.

Third, regarding causal inference assumptions, potential spatial spillover effects were not fully captured. In reality, residents of non-pilot cities may have traveled across regions to access more favorable sports consumption subsidies and superior sports infrastructure in neighboring pilot cities, thereby also benefiting from the policy dividends. This violation of the “stable unit treatment value assumption” implies that the control group was not purely unintervened. Future research integrating spatial econometric methods could more accurately quantify these cross-regional externalities and validate the persistence of these effects by extending the observation period.

### 5.2 Conclusion

The cost-containment effects of the SCC construction are not driven by a single factor but are achieved through four distinct, parallel mediating pathways. Specifically, the supportive environment created by the policy simultaneously produces the following four outcomes: (1) Increases sports consumption, redirecting household budgets from passive medical treatment toward proactive health investment; (2) Increases exercise frequency, physiologically slowing the depreciation of health capital; (3) Improves self-rated health, reducing unnecessary medical visits driven by health anxiety; (4) Reduces depression levels, mitigating somatic symptoms associated with psychological distress. These four independent channels synergistically transform residents' health management paradigm from passive reliance on medical services to proactive health maintenance. However, the distribution of these policy dividends exhibits structural heterogeneity, prompting critical reflection on health equity. The cost-containment effect on healthcare expenditures is most pronounced among seniors (aged 60+) and residents of central cities, indicating that accessible infrastructure amplifies the marginal benefits of health investments for high-risk groups. Conversely, this effect weakens for individuals burdened by mortgage debt and those with pre-existing chronic conditions. This suggests that financial liquidity is a prerequisite for engaging in sports consumption. Moreover, current policies function more effectively as primary prevention tools for healthy populations than as therapeutic interventions for those with existing conditions.

In summary, the SCC construction robustly validates the “exercise as medicine” hypothesis at the urban governance level, providing a replicable blueprint for developing countries to alleviate healthcare burdens through consumption-based upstream interventions. This study shows that public policies that foster active lifestyles can serve as a financially sustainable stabilizer for healthcare systems, effectively shifting health management from clinical settings to community environments. However, despite significant overall cost-control benefits, the study identified structural heterogeneity: policy dividends disproportionately favor financially secure and healthier individuals, highlighting risks of widening health disparities. Future policy iterations must therefore move beyond blanket promotion and embrace a new paradigm of “inclusive precision governance.” To maximize social welfare, policymakers should prioritize: (1) Introducing targeted sports subsidies or vouchers for liquidity-constrained households to lower participation barriers; (2) Institutionalizing “integrated medical and sports care,” such as formally incorporating “exercise prescriptions” into chronic disease management protocols. These measures are crucial to ensuring that the health dividends and economic efficiencies generated by such reforms are not merely aggregate gains but are equitably shared across all socioeconomic strata, ultimately realizing the vision of “health for all.”

## Data Availability

The raw data supporting the conclusions of this article will be made available by the authors, without undue reservation.
